# MEMORY FUNCTIONING AND CAREGIVING: CONTINUITY, CONNECTEDNESS AND METAPHORS IN ALASKA NATIVE PERCEPTIONS

**DOI:** 10.1093/geroni/igz038.3193

**Published:** 2019-11-08

**Authors:** Rosellen M Rosich, Maria Crouch

**Affiliations:** 1 University of Alaska Anchorage, Center for Human Development, Anchorage, Alaska, United States; 2 University of Alaska Anchorage, UAA-UAF PhD in Clinical-Community Psychology with a Rural and Indigenous Emphasis, Wasilla, Alaska, United States

## Abstract

Biomedical models often define dementia in a negative and diachronic manner, which shape Western, cultural understandings and approaches. However, utilizing a critical gerontological approach has allowed the current study to explore Alaska Natives (i.e., adults who hold “Elder” status and are 50 years or older) perception of memory decline, and the stresses imposed upon caregiving when a Western biomedical model of dementia is utilized. Multitudinous research demonstrates definitions and intersections of health, illness, ethnicity, and family are not universal. Subsequently, it is critical to examine these sociocultural concepts from diverse cultural belief systems and imperative to examine historical processes impacting these constructs to identify specific risk and protective factors regarding holistic health. Recent qualitative data analysis from an exploratory study of Alaska Native Elder’s perception of memory functioning and dementia has yielding themes that are consistent with previous research on indigenous culture. However, themes of continuity, connectedness, spirituality, intergenerational transmission, traditional belief systems, and barriers to cultural continuity such as oppression and historical trauma, are being filtered through Alaska Natives unique cultural lens. This cultural lens allows Alaska Natives to utilize positive metaphors for memory functioning and dementia embedded within their belief systems and these are distinct from Western biomedical definitions. The poster proposed will highlight themes recently uncovered from thematic analysis, code book development, and code matrices as well as present the positive, culturally adaptive and congruent representations that Alaska Native Elder’s utilize in understanding memory changes and forms of dementia that both explain and transcend biomedical models.

